# Nonunion of the Medial Cuneiform: A Rare Case

**DOI:** 10.1155/2013/215756

**Published:** 2013-07-30

**Authors:** Celil Alemdar, Bekir Yavuz Uçar, Azad Yıldırım, Ahmet Kapukaya

**Affiliations:** ^1^Department of Orthopaedics and Traumatology, Dicle University Medical Faculty, 21280 Diyarbakır, Turkey; ^2^Department of Orthopaedics and Traumatology, Seyrantepe Private Hospital, 21220 Diyarbakır, Turkey

## Abstract

Isolated medial cuneiform fractures are quite rare. Conservative treatment is adequate in most cases, while deplaced or unstable fractures are treated surgically. Nonunion is seen extremely rarely after medial cuneiform fractures. There is only one case report in the literature. This case presented here is a 62-year-old male patient who had an isolated medial cuneiform fracture resulting from the impact of a falling metal object. Conservative treatment was performed initially. The patient was diagnosed as nonunion after physical and radiological examinations nine months after he presented to the outpatient clinic. Internal fixation with a mini plate and one staple after reduction was performed surgically. Defective region was filled with a 2 mL of autograft, and the operation was terminated.

## 1. Introduction

 Medial cuneiform fractures are generally accompanied with ankle, cuboid, or tarsometatarsal joint injuries. Isolated fractures are rare. Cuneiform fractures accompanied with feet and ankle joint injuries generally develop due to axial loading and mediolateral or plantodorsal forces, while isolated cuneiform fractures occur as a result of direct trauma most of the time. Treatment of isolated fractures is based on maintaining the length of the foot while preserving associations of cuneiform with the surrounding structures. Although conservative treatment is generally satisfactory, surgery might be performed in irreducible or unstable fractures. Nonunion is a rarely seen complication of medial cuneiform fractures; only a single case is reported in the literature [1].

## 2. Case Report

**Figure 1:**
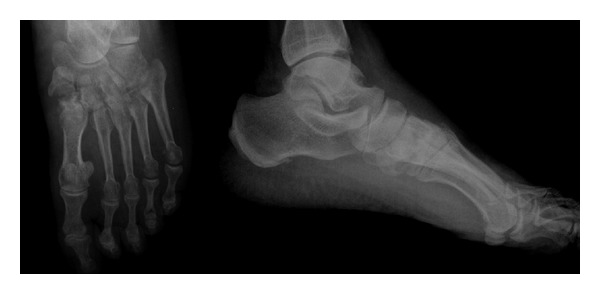
Preoperative anteroposterior and lateral X-ray.

**Figure 2:**
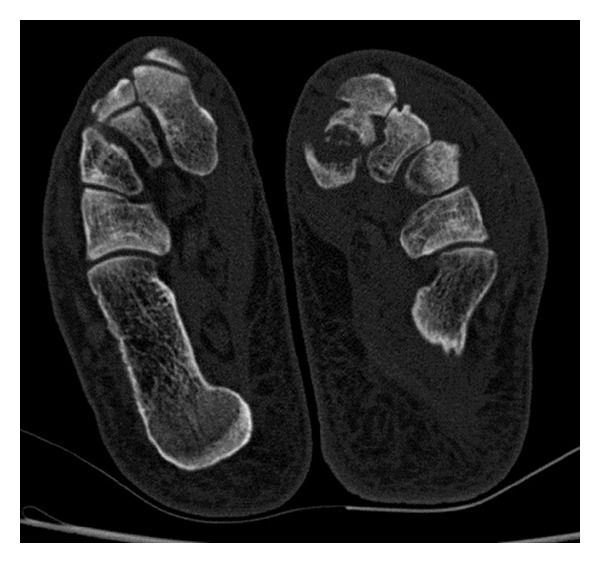
Preoperative computed tomography.

**Figure 3:**
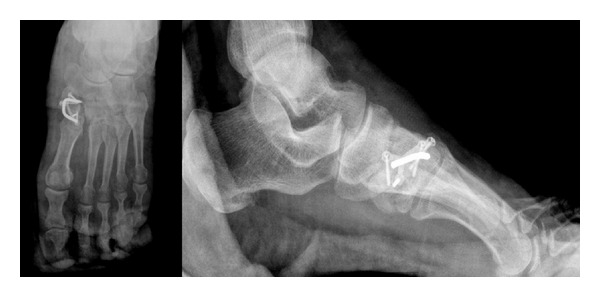
Postoperative anteroposterior and lateral direct X-ray.

 A 62-year-old male patient presented to our clinic with pain in the left foot with a limp. Swelling and tenderness were found to be present on medial cuneiform. His past medical history revealed a trauma nine months ago which had been treated conservatively. Anteroposterior (AP) direct X-ray of the left foot and direct lateral roentgenograms of the feet and a computed tomography were obtained (Figures 1 and 2). Sclerotic fracture tips and deplaced fracture fragments were interpreted as the nonunion of the medial cuneiform. Surgical treatment was planned. During surgery, a 4 cm longitudinal incision on medial cuneiform under regional anesthesia was performed, and fracture fragments were exposed. Sclerotic fracture tips and fibrous tissues were cleaned. After reduction was provided, osteosynthesis was performed with the use of 4 screws, one mini plate, and one staple. We used autograft to accelerate the healing. Spongiose autograft from the iliac crest in 2 mL was used to fill the defect between the fracture fragments. Postoperative antero-posterior and lateral direct roentgenograms were obtained (Figure 3).

## 3. Discussion

 Medial cuneiform fractures which are commonly a part of a rather complex injury affecting the foot and ankle joint rarely occur as an isolated injury [2]. The diagnosis is difficult due to the complex nature of the anatomic structures of the bones of the feet. Especially isolated nondisplaced fractures may be overlooked with a delay in the treatment of those patients [3]. Presence of a more complicated injury in the surrounding structures might masquerade the cuneiform fractures. 

 Three-way foot X-rays should be obtained for the diagnosis. When the direct roentgenograms are not adequate to mitigate the suspicions, computed tomography, magnetic resonance imaging, and bone scans might be used in the diagnosis [4]. Computed tomography is effective in evaluating the cortical structures, deplacement, and dislocation when direct roentgenograms are inadequate [5]. On the other hand, magnetic resonance imaging is more useful in the early diagnosis of fractures due to microtrauma [6]. 

 Isolated medial cuneiform fractures are commonly non-displaced and stable and thus can be treated conservatively with 6 to 8 weeks of immobilization with a short leg cast. Deplaced fractures, on the other hand, should be reduced and internally fixed to maintain the reduction when necessary [7]. In this way, the length of the foot can be maintained with a decreased rate of complications. 

 In cases with a suspicion of cuneiform fracture, bipartic medial cuneiform which is defined as a variant of the normal anatomy should also be considered in the differential diagnosis [8]. The direction and the plane of the fracture are important radiologically. Direction to coronal plane is usually present in fractures, while a more horizontal planar direction is found in bipartite medial cuneiform [9]. 

 Nonunion after isolated medial cuneiform fracture is an extremely rare complication. Only one case report was encountered during the literature review that we performed to search this entity. Deplacement of the fracture fragments, instability of the fracture line, and the soft tissue in between might have all contributed to the nonunion in our case. Nonunion may develop even in the fractures of the medial cuneiform which actually has a high tendency to union especially in fractures with deplaced fragments, in addition to the presence of other factors that pave the way to nonunion. 

 We recommend surgical therapy in medial cuneiform fractures, especially with high rate of deplacement or development of deplacement during followup.

## References

[1] Bryant MJ, Baird DS (1993). A case of non-union of the medial cuneiform. *Injury*.

[2] Cheng Y, Yang H, Sun Z, Ni L, Zhang H (2012). A rare midfoot injury pattern: navicular-cuneiform and calcaneal-cuboid fracture-dislocation. *Journal of International Medical Research*.

[3] Taylor SF, Heidenreich D (2008). Isolated medial cuneiform fracture: a special forces soldier with a rare injury. *Southern Medical Journal*.

[4] Olson RC, Mendicino SS, Rockett MS (2000). Isolated medial cuneiform fracture: review of the literature and report of two cases. *Foot and Ankle International*.

[5] Yutani Y, Ikeda K, Yamano Y (1996). Medical cuneiform fracture-dislocation associated with medial and intermediate cuneiform fractures. *Osaka City Medical Journal*.

[6] Khan KM, Brukner PD, Bradshaw C (1993). Stress fracture of the medial cuneiform bone in a runner. *Clinical Journal of Sport Medicine*.

[7] Patterson RH, Petersen D, Cunningham R (1993). Isolated fracture of the medial cuneiform. *Journal of Orthopaedic Trauma*.

[8] O’Neal ML, Ganey TM, Ogden JA (1995). Fracture of a bipartite medial cuneiform synchondrosis. *Foot and Ankle International*.

[9] Guler F, Baz AB, Turan A, Kose O, Akalin S (2011). Isolated medial cuneiform fractures: report of two cases and review of the literature. *Foot & Ankle Specialist*.

